# N-formylmethionine-leucyl-phenylalanine protects against irradiation-induced damage to hematopoiesis and intestines

**DOI:** 10.1186/s10020-024-00918-4

**Published:** 2024-09-10

**Authors:** Zhihua Li, Yatong Wu, Jicong Du, Wen Qian, Sinian Wang, Fengsheng Li, Suhe Dong, Shunchang Jiao

**Affiliations:** 1 Chinese PLA Medical School, Beijing, 100853 China; 2 The Postgraduate Training Base of Jinzhou Medical University (The PLA Rocket Force Characteristic Medical Center), Beijing, 100088 China; 3 Department of Radiation Medicine, Faculty of Naval Medicine, Naval Medical University, Shanghai, 200433 China; 4 Department of Nuclear Radiation Injury and Monitoring, The PLA Rocket Force Characteristic Medical Center, Beijing, 100088 China; 5 PLA Rocket Force Characteristic Medical Center, Beijing, 100088 China; 6 Department of Oncology, the First Medical Centre, Chinese PLA General Hospital, Beijing, 100853 China

**Keywords:** fMLP, Ionizing radiation, Cell cycle arrest, Intestinal injury

## Abstract

**Background:**

Ionizing radiation (IR), including radiotherapy, can exert lasting harm on living organisms. While liposaccharide (LPS) offers resistance to radiation damage, it also induces toxic responses. Thankfully, an LPS analogue called N-formylmethionine-leucyl-phenylalanine (fMLP) holds the potential to mitigate this toxicity, offering hope for radiation protection.

**Methods:**

Survival of C57BL/6 mice exposed to IR after administration with fMLP/LPS/WR-2721 or saline was recorded. Cell viability and apoptosis assay of bone marrow (BMC), spleen and small intestinal epithelial (HIECs) cells were tested by Cell Counting Kit-8 (CCK-8) and flow cytometry assay. Tissue damage was evaluated by Hematoxilin and Eosin (H&E), Ki-67, and TUNEL staining. RNA sequencing was performed to reveal potential mechanisms of fMLP-mediated radiation protection. Flow cytometry and western blot were performed to verify the radiation protection mechanism of fMLP on the cell cycle.

**Results:**

The survival rates of C57BL/6 mice exposed to ionizing radiation after administering fMLP increased. fMLP demonstrated low toxicity in vitro and in vivo, maintaining cell viability and mitigating radiation-induced apoptosis. Moreover, it protected against tissue damage in the hematopoietic and intestinal system. RNA sequencing shed light on fMLP’s potential mechanism, suggesting its role in modulating innate immunity and cell cycling. This was evidenced by its ability to reverse radiation-induced G2/M phase arrests in HIECs.

**Conclusion:**

fMLP serves as a promising radioprotective agent, preserving cells and radiosensitive tissues from IR. Through its influence on the cell cycle, particularly reversing radiation-induced arrest in G2/M phases, fMLP offers protection against IR’s detrimental effects.

## Introduction

The advancement of nuclear technology offers remarkable economic gains, but a shadow of concern arises from the increasing occurrence of radiation-induced health issues (Yang et al. 2016; Prăvălie and Bandoc 2018). The intricate complexities of radiation exposure, particularly during cancer radiotherapy, emphasize the critical need for protective strategies against its harmful effects on healthy bodily tissues (Ghita et al. 2019; Wei et al. 2019). The urgency for developing novel radioprotective agents is underscored by the realization that severe radiation exposure leads to multifaceted physiological disruptions, affecting multiple systems including the nervous, digestive, and hematological apparatus. Among the myriad challenges posed by irradiation, the intricate damage to the intestinal epithelium stands out, involving phenomena such as cell demise, stem cell impairment, immune responses, and nutrient malabsorption. These complex mechanisms highlight the necessity for focused interventions, especially in vulnerable tissues like the intestine, to mitigate these challenges and enhance therapeutic outcomes (Xie et al. 2020). The quest for novel radioprotective strategies is thus paramount to achieving meaningful advancements in clinical practice.

Recent research has unveiled a fascinating potential for bacteria-derived compounds, such as lipopolysaccharide (LPS), in enhancing the body’s resilience against radiation damage and mitigating cell apoptosis induced by this stressor (Liu et al. 2018; Hegyesi et al. 2019). LPS, an integral constituent of Gram-negative bacterial cell walls, holds the key to activating the innate immune response for radioprotection (Simpson and Trent 2019). Despite exhibiting anti-radiation properties, LPS can also induce severe systemic toxicity (Cavaillon 2018). Our earlier study hinted at a promising candidate—the leukocyte chemotactic peptide fMLP, an analog of LPS—which elicits comparable inflammatory reactions, thus hinting at its potential radioprotective role (Yang et al. 2008). However, the feasibility of employing fMLP as a radioprotective agent remains unexplored terrain.

In this study, we confirmed that fMLP offered radioprotection for both hematopoietic and intestinal systems, with in vivo and in vitro efficacy. Moreover, unraveling the intricate mechanisms behind fMLP’s radioprotective attributes. These novel insights into the realm of radioprotection open up exciting avenues for future exploration and innovation. The quest to harness these protective strategies against radiation damage continues apace, holding the promise of significant advancements in clinical settings.

## Materials and methods

### Chemicals and reagents

fMLP (CAS No.: 59880-97-6) was purchased from Sigma-Aldrich (Merck, United States). Normal saline (NS) was obtained from ChangHai Hospital (Shanghai, China). The apoptosis detection kit was purchased from Transgen (Beijing, China), and RPMI1640 and fetal bovine serum were supplied by Gibco (United States). PBS, Chloroform, Isopropyl alcohol, and 75% ethanol (75% C_2_H_5_OH) were obtained from Shanghai Bio-Light Biotech Corporation (Shanghai, China). Cell Counting Kit-8 (CCK-8) was purchased from Dojindo (Kumamoto, Japan). Tissue fixation solution, decalcification solution, and hematoxylin-eosin staining solution were purchased from Wuhan Google Biotechnology Company (Wuhan, China). Trizol reagent was supplied by Invitrogen (United States). Methanol was obtained from the Beijing Chemical Plant (Beijing, China). PCR reagents were supplied by Dalian BMG (Dalian, China).

### Cell culture and treatment

A human small intestinal epithelial cell line (HIEC) was purchased from ATCC (American Type Culture Collection, USA). HIEC culture conditions were RPMI 1640 (Gibco, United States) with 10% fetal bovine serum and 1% Penicillin-Streptomycin (Gibco, United States) and 5% CO_2_. The cells were cultured in an incubator with constant temperature and humidity at 37℃.

### Animals and treatment

6–8 weeks C57BL/6 male mice were purchased from the Shanghai Chinese Academy of Sciences and kept in the animal room of Naval Medical University. Feeding environment: indoor ventilation, 24℃-26℃, strict regulation of 12 h of light day and night, free intake of food and water. Mouse husbandry and all animal experimental procedures were approved by the PLA Rocket Force Characteristic Medical Center for Specialty Medicine and performed following the National Institutes of Health Guide for the Care and Use of Laboratory Animals (KY2020038). Forty-two C57BL/6 male mice were divided into seven groups and treated with different doses of fMLP, LPS, or WR-2721 (*n* = 6), the survival rate of the mice was observed and recorded. 120 mice were randomly divided into control, fMLP, LPS, and WR-2721 groups. After administration, the mice were irradiated with γ-rays at 7.5 Gy, 9 Gy, and 12 Gy, and then the survival of mice in each group (*n* = 10) was observed. To verify the effect of fMLP on peripheral blood leukocytes of irradiated mice, after whole-body irradiation, the peripheral blood leukocytes were measured and detected in the fMLP group and the control group by dividing into six groups at 1, 5, 10, 15, 20, and 30 days (*n* = 3 biological replicates). The assessment of intestinal pathological stains was performed on days 0, 1, 3, and 7 after 10 Gy irradiation (*n* = 3 biological replicates).

### Irradiation

^60^Co source in the radiation center (Faculty of Naval Medicine, Naval Medical University, China) was used to irradiate mice and cells. The rate of irradiation was 1 Gy/min.

### CCK-8 assay

To evaluate the toxicity of fMLP and cell viability, the CCK-8 assays were utilized. Cells were seeded at 5.0 × 10^3^ cells per well into the flat-bottomed 96-well plates with 100 μl culture medium. Then, 10 μl of CCK-8 solution (Dojidon, Kumamoto, Japan) was added to the wells, and incubated at 37 °C in a 5% humidified CO_2_ atmosphere. A microplate reader (Thermo Fisher, United States) was employed to measure the absorbance of samples at 450 nm.

### Antibody staining and flow cytometry

Bone marrow cells (BMCs) were isolated freshly. Then cells were strained through a 40 μm strainer in the presence of PBS and red blood cells were removed. Cells were stained with antibody for 20 min at 4 ℃. After collecting the cells and giving them twice PBS washes, 500 μL of binding buffer, 5 uL of membrane-bound protein Annexin V-FITC, and 5 μL of PI-PE staining solution were added to the cells for fluorescence labeling. The cells were then left at room temperature and shielded from light for 15 min. Using flow cytometry, observation and detection were carried out. The cell apoptosis and cell cycle were analyzed by the apoptosis and cell cycle detection kits according to the manufacturer’s instructions.

### RNA extraction for RNA sequencing analysis

After the primary spleen cells were collected by centrifugation, an appropriate amount of Trizol (50–100 mg/ml) was added for repeated absorption and beating to 1.0 ml. Then homogenate samples were placed at room temperature (15–30℃) for 5 min, and nucleic acid-protein complexes were completely isolated. The 0.2 ml chloroform was added to 1.0 ml Trizol, then the samples were shaken for 15 s and placed at room temperature for 3 min. Centrifuge at 10,000 g for 15 min at 2–8℃. Transfer the aqueous phase to the new tubes, and then use isopropanol to precipitate the aqueous phase RNA. Each 1.0 ml Trizol was added with 0.5 ml isopropanol and left at room temperature for 10 min. Centrifugation at 10,000 g for 10 min at a temperature of 2–8℃, then 75% ethanol washed RNA precipitation. After centrifugation at 7500 g for 5 min at 2–8℃, the supernatant was discarded. Dry the RNA precipitation for 5–10 min at room temperature. Add 25–200 μl RNase-free water for RNA dissolution. RNA-seq analysis, after extracting total RNA from the samples, the RNA was quantified and quality controlled using a bioanalyzer. The fragmented RNA transcripts were ligated with aptamers to construct a library. The RNA fragments in the library were subjected to high-throughput sequencing using the Illumina platform.

### Western blot assay

HIEC cells were inoculated in 6 well plates at an appropriate cell density (1 × 10^6^ cells), and after treatment, the cells were collected, and the appropriate amount of RIPA lysis solution was added according to the number of cells, and the cells were lysed on ice for 30 min. After sufficient lysis, the cells were centrifuged at a temperature of 4 °C for 15 min, then the supernatant was taken. The absorbance value of the protein solution at 562 nm was determined, and the protein concentration of each sample was converted using the protein standard curve. Equal amounts of protein were subjected to electrophoresis (150 V, 50 min). Separated proteins were transferred onto polyvinylidene fluoride membranes (200 mA, 2 h). The membranes were blocked with 5% defatted milk in Tris Buffered saline Tween (TBST) for 1 h at room temperature. The membrane was added with diluted primary antibody (1:1000) and incubated at 4 °C overnight. At room temperature, the incubated membrane was washed three times with TBST. The diluted secondary antibody solution (1:5000) was added and incubated for 1 h at room temperature. Chemiluminescence was captured on gel imaging system (BioRad ChemiDoc MP).

### Statistical analysis

GraphPad Prism 8 was used to draw and analyze statistical data in this study. Experimental data were expressed in the form of mean ± standard errors (SEM). The samples were normally distributed between the two groups. Unpaired t-test was used with equivariance, and Unpaired t-test with Welch’s correction was used with equivariance. Univariate analysis of variance (ANOVA) was used to analyze the differences between 3 groups and more (consistent with normal distribution and homogeneous variance), and further pair-Whitney test was used for pairwise comparison. Kaplan-Meier method was used to analyze the difference in survival time between the two groups. All experiments were repeated at least three times. *p* < 0.05 was considered statistically significant.

## Result

### fMLP behaved low toxicity in cells and mice

**Fig. 1:**
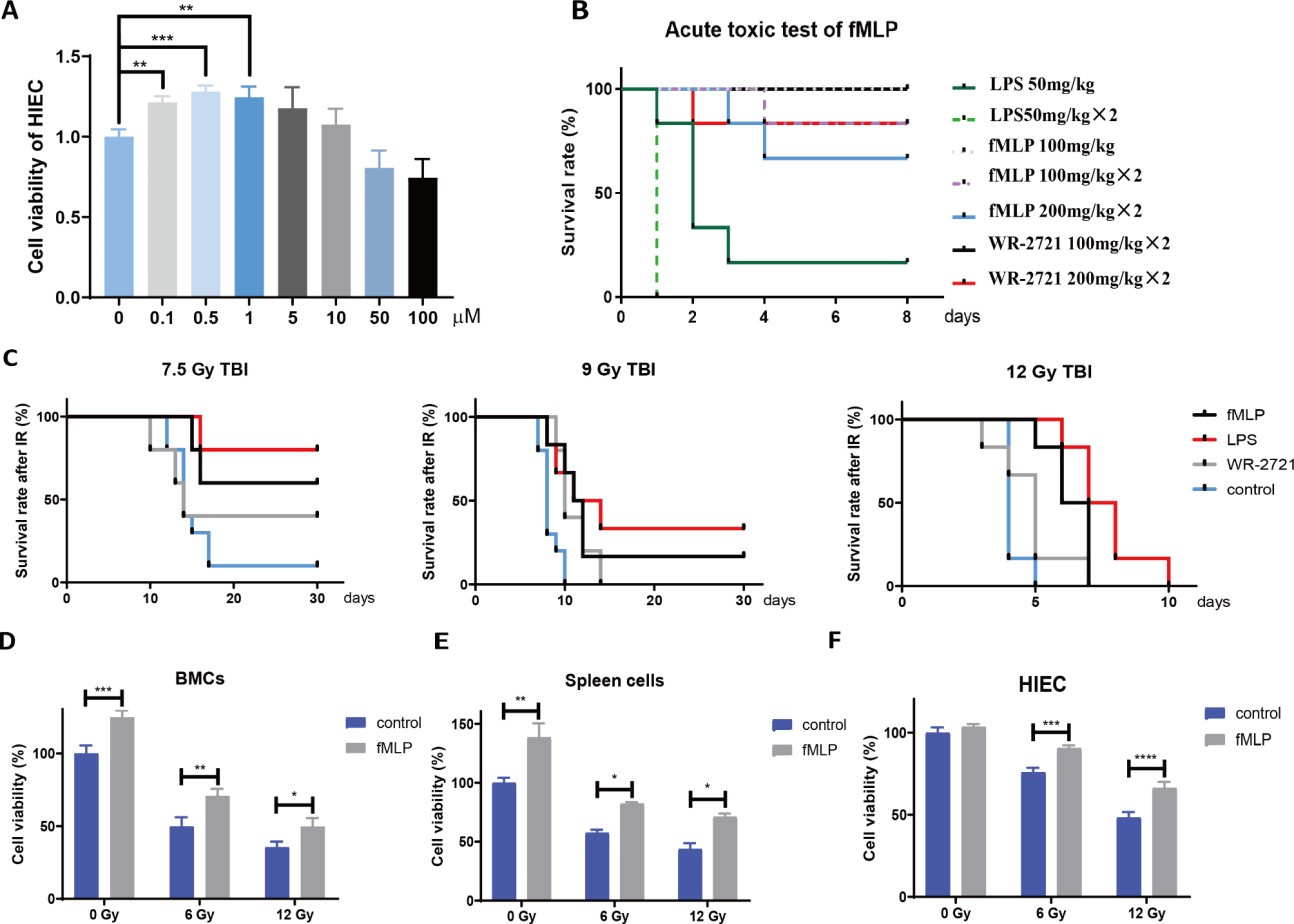
fMLP behaves low toxicity and radioprotection in cells and mice. (**A**) The cell toxicity of fMLP and viability detection in HIEC. The cells were treated by fMLP according to a certain concentration gradient dose for 24 h, then the cell viability was detected by the CCK-8 analysis. (**B**) The acute toxicity test of LPS, WR-2721, and fMLP in mice. C57BL/6 mice were treated with different reagents and their 10-day survival period was recorded. (*n* = 6). (**C**) fMLP has a certain radiation protection effect in vivo. The mice were randomly divided into a control group, fMLP group, LPS group, and WR-2721 group. After administration, mice were irradiated with 7.5 Gy, 9 Gy, and 12 Gy gamma rays, then the survival of mice in each group was observed. (*n* = 10). (**D-F**) fMLP has a certain radiation protection effect in vitro. HIEC, BMCs, and spleen cells in the administration group were stimulated with fMLP 12 h and 2 h before IR, followed by irradiation with different doses (6.0 Gy and 12.0 Gy), and changes in cell viability in the fMLP group and control group were detected 24 h after irradiation. Data are expressed as mean ± SEM, * *p* < 0.05

To thoroughly evaluate the safety profile of fMLP, a comprehensive in vitro and in vivo assessment was conducted. In vitro, human small intestinal epithelial cells (HIECs) were exposed to varying concentrations of fMLP, and cell viability was determined using the CCK-8 assay. The results revealed a dose-dependent relationship between fMLP concentration and HIEC viability, with 0.5 μM emerging as the optimal concentration that supported cellular viability (Fig. 1A). For in vivo evaluation, Forty-two C57BL/6 mice were divided into seven groups receiving different doses of fMLP, LPS, or WR-2721 (a well-established radioprotective agent) via intraperitoneal (*i.p*) injections. The survival rates of mice were meticulously monitored and recorded over a 10-day period post-injection. Notably, a remarkable survival rate of 100% was observed in the group receiving 100 mg/kg of fMLP (*p* < 0.0001), while half of the mice remained viable even with a doubling dose of 200 mg/kg×2. In stark contrast, LPS demonstrated significantly higher toxicity, with a mere 20% survival rate at 50 mg/kg, and no survivors at a doubling of the dose to 50 mg/kg×2. Interestingly, fMLP exhibited comparable safety to the established radioprotective agent WR-2721, as there were no significant differences in survival rates between these two groups (Fig. 1B). These findings provide compelling evidence suggesting that fMLP possesses a promising safety profile, both in vitro and in vivo, which holds encouraging implications for its potential application in radioprotection.

### fMLP has a radioprotective effect in vitro and in vivo

The radioprotective potential of fMLP was investigated in both in vitro and in vivo settings, revealing promising outcomes (Fig. 1A-B). A remarkable increase in survival rates was observed following fMLP administration compared to the control group post-irradiation exposure (Fig. 1C). The radioprotective effect of LPS was equivalent to fMLP, and was better than that of the WR-2721 group. At the doses of 7.5 and 9.0 Gy, there was almost no difference between the WR-2721 group and the control group, while statistical differences appeared in LPS and fMLP group. Notably, the survival rate of mice subjected to irradiation in both the LPS group and fMLP group was significantly enhanced at an irradiation dosage of 12 Gy, as compared to the WR-2721 control group. Specifically, the Kaplan-Meier survival curve analysis revealed a marked improvement in overall survival probability for mice treated with LPS or fMLP prior to irradiation, with a corresponding increase in median survival time (Fig. 1C). To unravel the underlying protective mechanism of fMLP, three distinct cellular lineages—HIECs, bone marrow cells (BMCs), and spleen cells—were chosen for evaluation. These cells were divided into control and fMLP groups, with the latter receiving a 0.5 μM dose of fMLP prior to irradiation. The CCK-8 assay was employed 24 h post-irradiation with doses of 6.0 and 12.0 Gy, revealing a significant reduction in cell viability, which was remarkably mitigated upon fMLP administration for all three cell types following both radiation strengths (Fig. 1D and F). Above all, fMLP offers robust radioprotection across diverse cellular contexts, suggesting its potential as a promising radioprotective agent.

### Radiation protection effect of fMLP on the hematopoietic system

**Fig. 2:**
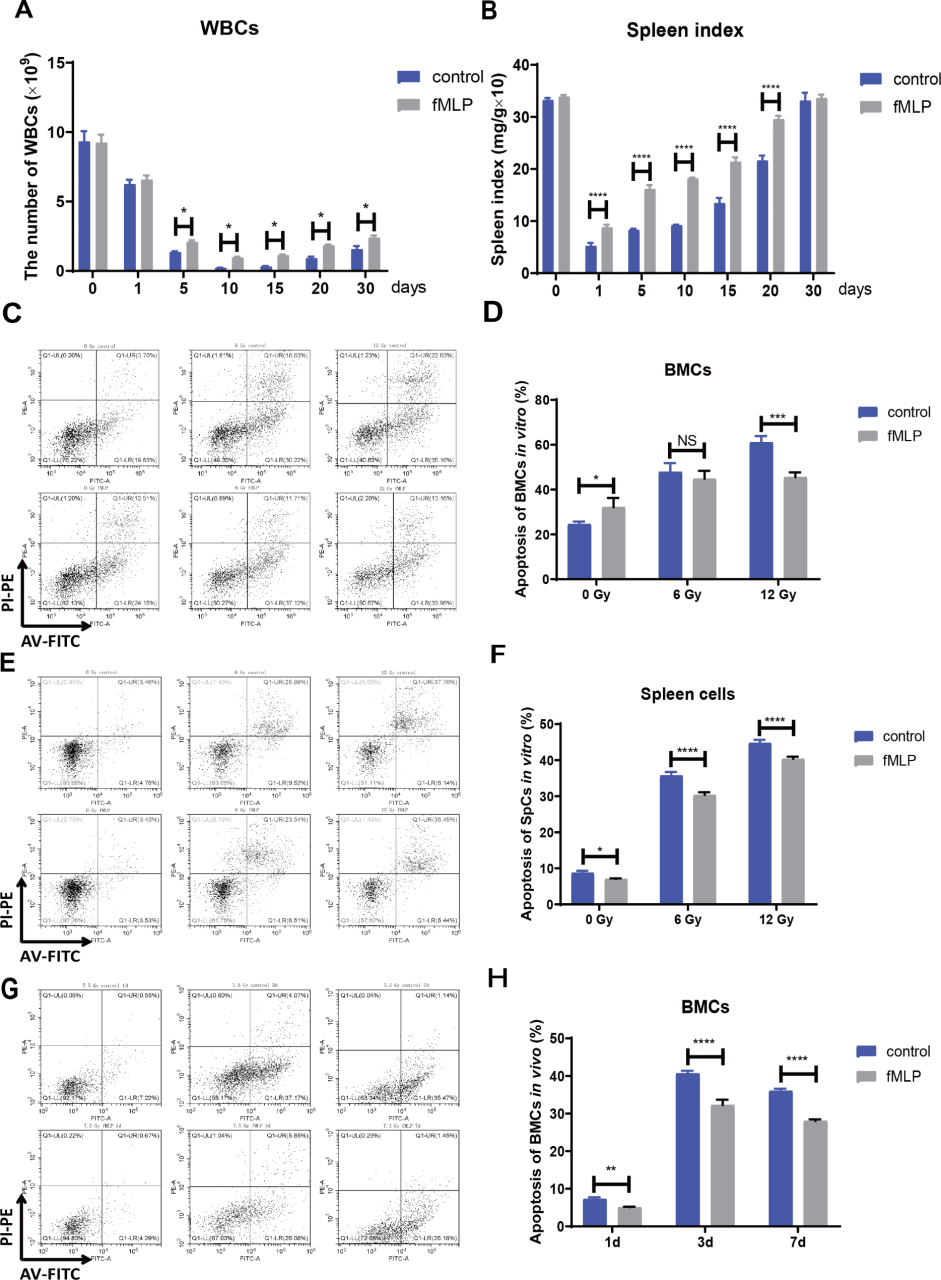
fMLP has a protective effect on the damage of the mouse hematopoietic system induced by irradiation. (**A**) fMLP increased the value of peripheral blood WBCs in mice after irradiation. After 6 Gy total-body irradiation, the peripheral blood WBCs were measured on the 1, 5, 10, 15, 20, and 30 days (*n* = 3 biological replicates). (**B**) fMLP increased the spleen index of mice after irradiation. After whole-body irradiation, the mouse spleens were taken to calculate the spleen organ index of each group on the 1, 5, 10, 15, 20, and 30 days (*n* = 3 biological replicates). (**C**) fMLP alleviated the apoptosis of bone marrow cells in vitro after irradiation. The mice in the fMLP group were given 24 h and 2 h before the irradiation, and the BMCs in vitro were analyzed after 6.0 Gy and 12.0 Gy γ-ray irradiation for apoptosis by flow cytometry (*n* = 3 biological replicates). (**D**) Statistical graph of BMCs apoptosis in vitro. (**E**) fMLP alleviated the apoptosis of spleen cells in vitro after irradiation. (F) Statistical graph of spleen cells apoptosis in vitro. (**G**) fMLP decreased BMCs apoptosis in vivo after γ-ray irradiation. Mice were given fMLP stimulation 12 h and 2 h before 7.5 Gy irradiation, and BMCs were extracted 1,3, and 7 days after irradiation to detect apoptosis by flow cytometry (*n* = 3). (**H**) Statistical graph of BMCs apoptosis rate in vivo. Data are expressed as mean ± SEM, * *p* < 0.05

Further, to explore the radioprotective impact of fMLP on the hematopoietic system, white blood cell (WBC) counts were obtained from peripheral blood scollected at various time points post-6 Gy -irradiation (1, 5, 10, 15, 20, and 30 days). Results revealed a significant decline in WBC counts following irradiation, with the lowest count observed between days 10 and 15. Notably, fMLP treatment yielded moderately lower WBC counts compared to the control group, showcasing a quicker recovery by day 5, suggesting its ability to alleviate IR-induced leukocyte depletion (Fig. 2A). Furthermore, spleen weight analysis revealed a notable difference in spleen index between the fMLP and control groups post-6 Gy-IR (1, 5, 10, 15, 20, and 30 days), indicating fMLP’s protective effect on the hematopoietic system (Fig. 2B). Additionally, flow cytometry analysis confirmed that fMLP diminished the IR-induced increase in apoptosis rates of bone marrow cells (BMCs) and spleen cells, providing direct evidence of its radioprotective role (Fig. 2C-F). In a separate experiment, C57BL/6 mice were administered fMLP at a dose of 1 mg/kg, 24 and 2 h prior to IR. BMCs were collected on days 1, 3, and 7 post-irradiation, revealing significantly lower apoptosis rates in the fMLP group compared to the control group (Fig. 2G-H). Collectively, fMLP exerts a protective effect on the hematopoietic system, mitigating IR-induced damage.

### The radioprotective effect of fMLP on the intestinal system

**Fig. 3:**
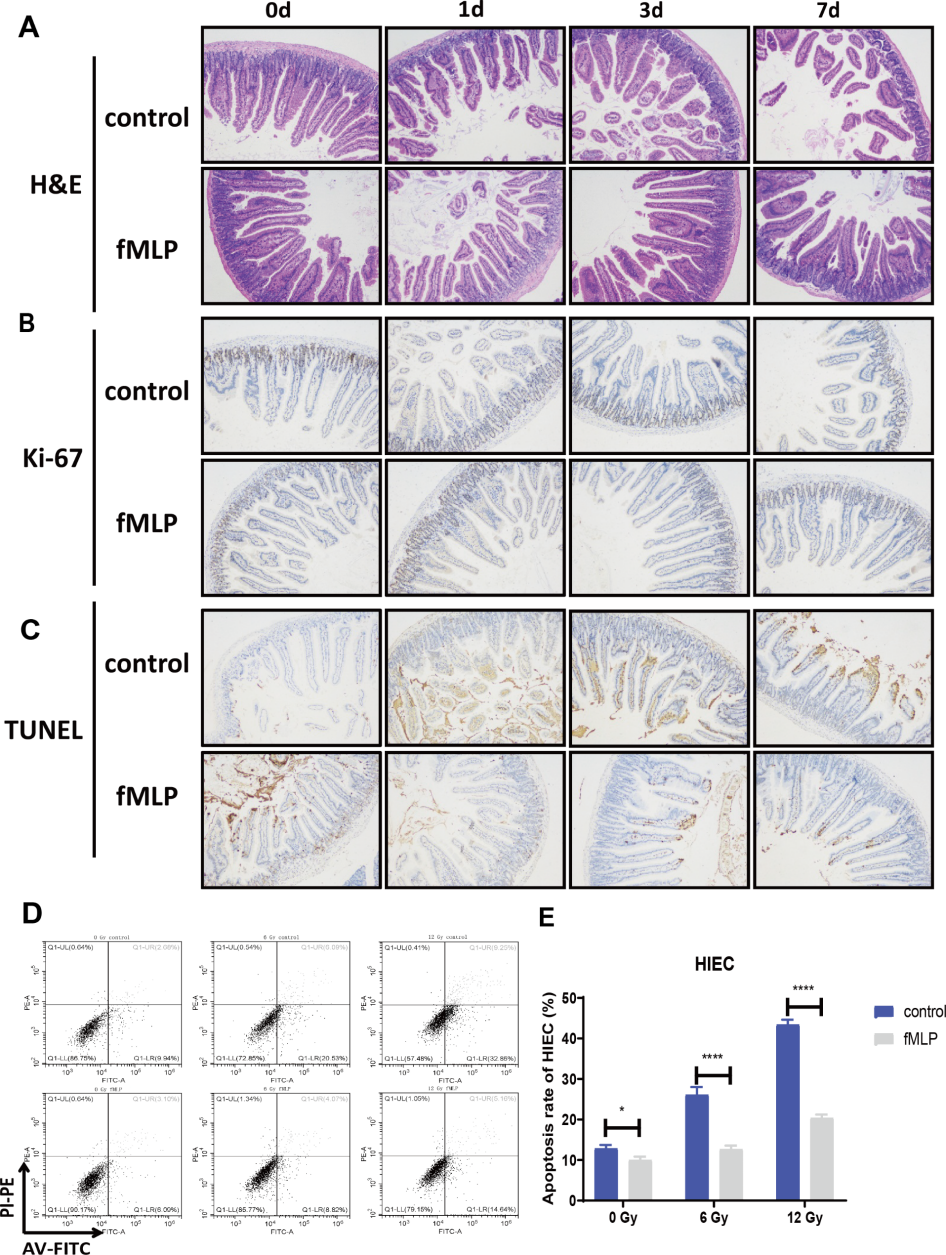
fMLP has a significant radiation protection effect on the mouse intestinal system. The intestines were collected after 10 Gy irradiation for pathological staining on 0, 1, 3, and 7 days (*n* = 3). The microscope magnification is 100×. (**A**) Intestinal H&E staining. (**B**) Intestinal Ki-67 staining. (**C**) Intestinal TUNEL staining. (**D**) fMLP was administrated 12 h and 2 h before irradiation, and the positive rates of apoptosis (6.0 Gy and 12.0 Gy) were detected by flow cytometry 24 h after irradiation. (**E**) Statistical graph of apoptosis rate of HIEC cell line. Data are expressed as mean ± SEM, * *p* < 0.05

A further exploration of fMLP’s radioprotective effects on intestinal tissue was conducted using C57BL/6 mice divided into control and fMLP groups. Mice in the fMLP group received doubling doses of fMLP at 1 mg/kg, 24 and 2 h prior to IR. Intestinal samples were collected for analysis at various time points post-irradiation (0, 1, 3, and 7 days). Hematoxylin and Eosin (H&E) staining revealed that intestinal damage was notably reduced in the fMLP group compared to the control group, particularly in terms of crypt numbers on days 3 and 7 (Fig. 3A). Ki-67 staining demonstrated a higher positive rate in the fMLP group, suggesting enhanced regeneration of intestinal crypt stem cells (Fig. 3B). TUNEL staining revealed a lower apoptosis rate in the fMLP group, indicating its radioprotective impact on intestinal tissue (Fig. 3C). Moreover, flow cytometry analysis confirmed that fMLP treatment diminished the IR-induced increase in the apoptosis rate of intestinal epithelial cells, providing further evidence of its protective role (Fig. 3D-E). Overall, fMLP exerts a radioprotective effect on intestinal tissue, promoting cellular regeneration and reducing apoptosis following IR.

### RNA-seq reveals the possible mechanism of fMLP as a radioprotectant

**Fig. 4:**
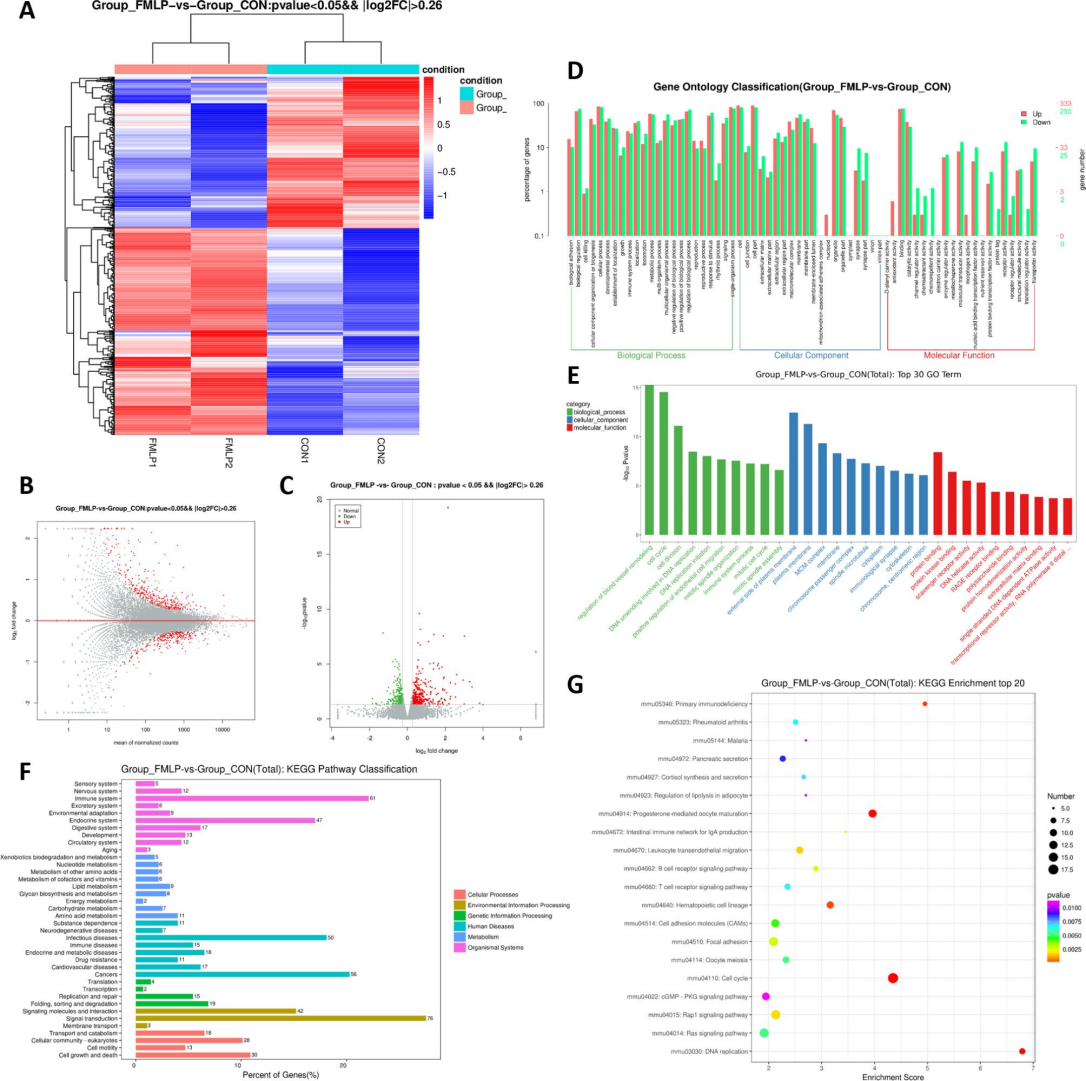
RNA-seq analysis of DEGs between the control and the fMLP group after IR. (**A**) DEGs clustering map (343 **u**p-regulated genes and 250 down-regulated genes, red is up-regulated gene, green is down-regulated gene). (**B**) DEGs MA map (Above the red line are up-regulated expressed genes; below the red line are down-regulated expressed genes). (**C**) DEGs volcano map (red is up-regulated expressed genes; green is down-regulated expressed genes). (**D**) The DEGs genes control group and fMLP group were analyzed by GO analysis. (**E**) Representative TOP 30 GO Terms. (**F**) KEGG analysis of DEG genes between the control group and the fMLP group. (**G**) Analysis of DEGs-enriched gene KEGG TOP 20. The ordinate axis is the channel name, and the abscissa is the enrichment factor. The larger the dot and the darker the color, the greater the enrichment factor

To gain insights into the underlying molecular mechanisms, RNA sequence analysis was performed using spleens collected from fMLP-treated and control mice 24 h after 7 Gy-irradiation. A total of 593 differentially expressed genes were identified, comprising 343 up-regulated and 250 down-regulated genes. Volcano and clustering heatmaps visually displayed these differentially expressed genes (Fig. 4A-C). Gene Ontology (GO) analysis categorized the differentially expressed genes into biological processes, cellular components, and molecular functions (Fig. 4D). The top hits for cellular components were the plasma membrane and cytoskeleton, while cell cycle and cell division were predominantly enriched in biological processes. Meanwhile, protein binding and protein kinase binding dominated the molecular function category (Fig. 4D). Notably, the cell cycle was among the most significantly enriched GO terms (Fig. 4D-E). Kyoto Encyclopedia of Genes and Genomes (KEGG) pathway enrichment analysis confirmed that the cell cycle pathway was the most significantly enriched, indicating its critical role in fMLP’s radioprotection (Fig. 4F-G). Tables showcasing the top GO terms (Top 30) and KEGG pathways (Top 20) in detail (Tables 1 and 2). Collectively, fMLP’s radioprotective effects are closely linked to cellular responses, primarily involving the regulation of the cell cycle and cytoskeletal organization.

**Table 1:** fMLP group vs. control group (total): the TOP 30 GO term

ID	Term	Category	ListHits	pval	Enrichment score
GO:0007049	cell cycle	biological process	53	1.45E-24	5.342425217
GO:0051301	cell division	biological process	36	7.55E-19	6.009009009
GO:0006270	DNA replication initiation	biological process	8	6.92E-11	20.6976977
GO:0006260	DNA replication	biological process	15	1.11E-10	7.697490879
GO:0006268	DNA unwinding involved in DNA replication	biological process	5	1.15E-10	44.35220935
GO:0000278	mitotic cell cycle	biological process	14	2.11E-10	8.04910466
GO:0007059	chromosome segregation	biological process	12	2.76E-09	8.099099099
GO:0007052	mitotic spindle organization	biological process	8	3.20E-09	14.19270699
GO:0000070	mitotic sister chromatid segregation	biological process	7	7.08E-09	16.09820932
GO:0002250	adaptive immune response	biological process	14	2.32E-08	5.756975519
GO:0042555	MCM complex	cellular component	6	9.24E-12	41.3953954
GO:0000775	chromosome, centromeric region	cellular component	16	2.23E-10	6.804722531
GO:0005876	spindle microtubule	cellular component	9	2.43E-10	15.10372535
GO:0032133	chromosome passenger complex	cellular component	4	1.05E-09	49.67447447
GO:0001772	immunological synapse	cellular component	8	2.41E-09	14.61013955
GO:0009897	external side of plasma membrane	cellular component	22	2.56E-09	4.26890015
GO:0072686	mitotic spindle	cellular component	10	9.22E-09	9.267625835
GO:0005856	cytoskeleton	cellular component	45	3.48E-08	2.342153553
GO:0005819	spindle	cellular component	10	4.15E-08	8.064038064
GO:0000776	kinetochore	cellular component	12	9.72E-08	6.057862741
GO:0005515	protein binding	molecular function	118	1.74E-07	1.519806054
GO:0003678	DNA helicase activity	molecular function	5	1.78E-07	18.26267444
GO:0019901	protein kinase binding	molecular function	24	7.63E-07	2.95095888
GO:0008017	microtubule binding	molecular function	13	1.04E-05	3.772010328
GO:0043142	single-stranded DNA-dependent ATPase activity	molecular function	3	1.99E-05	16.93447993
GO:0031994	insulin-like growth factor I binding	molecular function	3	4.21E-05	14.32917533
GO:0003688	DNA replication origin binding	molecular function	3	4.21E-05	14.32917533
GO:0003756	protein disulfide isomerase activity	molecular function	3	0.0002115	9.804172594
GO:0035174	histone serine kinase activity	molecular function	3	0.0002115	9.804172594

**Table 2:** fMLP group vs control group (total): KEGG enrichment TOP 20

ID	Term	ListHits	pval	Enrichment score
mmu04110	Cell cycle	18	2.30E-08	4.349417
mmu03030	DNA replication	8	1.59E-06	6.793375
mmu04914	Progesterone-mediated oocyte maturation	12	8.62E-06	3.962802
mmu05340	Primary immunodeficiency	6	0.000163	4.953502
mmu04640	Hematopoietic cell lineage	10	0.000296	3.16181
mmu04670	Leukocyte transendothelial migration	10	0.001637	2.584436
mmu04015	Rap1 signaling pathway	15	0.00179	2.133087
mmu04662	B cell receptor signaling pathway	7	0.002746	2.889543
mmu04510	Focal adhesion	14	0.002866	2.090926
mmu04672	Intestinal immune network for IgA production	5	0.002938	3.455932
mmu04514	Cell adhesion molecules (CAMs)	12	0.004297	2.12293
mmu04114	Oocyte meiosis	9	0.005248	2.325992
mmu04014	Ras signaling pathway	15	0.005269	1.91337
mmu05323	Rheumatoid arthritis	7	0.006593	2.506592
mmu04660	T cell receptor signaling pathway	8	0.006779	2.35414
mmu04927	Cortisol synthesis and secretion	6	0.007013	2.661583
mmu04972	Pancreatic secretion	8	0.008687	2.264458
mmu04923	Regulation of lipolysis in adipocyte	5	0.009994	2.70191
mmu05144	Malaria	5	0.009994	2.70191
mmu04022	cGMP - PKG signaling pathway	11	0.011041	1.946019

### fMLP reversed cell cycle arrest to protect from radiation damage

**Fig. 5:**
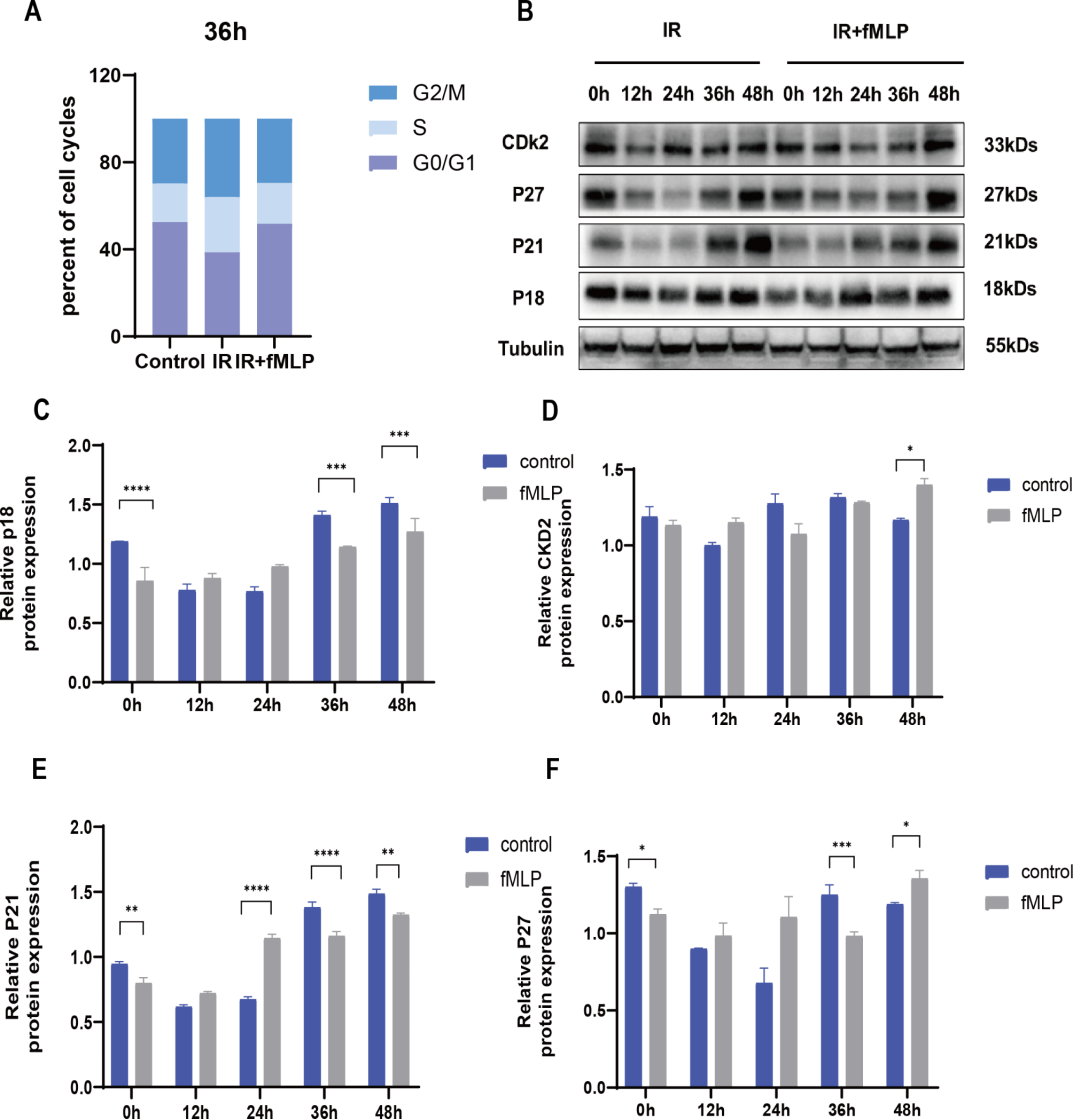
fMLP reverses cell cycle arrest and prevents radiation damage. (**A**) Analysis by flow cytometry for HIEC in different groups. (**B-G**) Treatment of HIEC cell cycle-related protein expression with fMLP administered after irradiation. The effects of fMLP on HIEC cell cycle-related protein expression were detected in irradiation alone and post-irradiation administration, respectively. Data are expressed as mean ± SEM, * *p* < 0.05

36 h after irradiation, fMLP treatment significantly increased HIEC cells in the S-phase (1.42 times, *p* < 0.05) and G2/M-phase (1.24 times, *p* < 0.05), while control cells predominantly remained in the G0/G1 phase(Fig. 5A), which indicated that fMLP promoted cell survival by facilitating cell cycle progression. Irradiation typically induces cell cycle arrest at G1/S and G2/M phases, with the latter being the most sensitive to IR. The cell cycle regulators p18, p21, and p27 are crucial in this process (Maddika, et al. 2007; Roy and Banerjee 2015; Lai et al. 2020). fMLP treatment notably downregulated the expression of these proteins prior to irradiation, enabling cells to reside in the G0/G1 phase (Fig. 5B-F). However, after IR exposure, fMLP treatment diminished the upregulation of p21, p27, and CDK2, thereby allowing cells to transition from the G0/G1 phase to the radio-sensitive G2/M phase (Fig. 5B-F). Thus, fMLP’s radioprotection involves manipulating cell cycle dynamics, specifically alleviating IR-induced cell cycle arrest in the G2/M phase by modulating the expression of cell cycle regulators.

## Discussion

Innate immunity is modulated by fMLP, as evidenced by multiple studies (Kilic et al. 2015; Wille et al. 2018). Specifically, it facilitates chemotaxis of leukocytes and macrophage activation (Polesskaya et al. 2014), contributing to a robust immune response. fMLP acts on neutrophils via a specific receptor called FPR (formylated peptide receptor), which belongs to the G-protein coupled receptor (GPCR) family, and mediates several signaling pathways, such as the PI3K-AKT pathway, the Rac-p38MAPK pathway, and the Ras-ERK1/2 pathway (Bedouhène et al. 2020). MEK1-ERK1/2 and p38MAPkinase pathways are associated with the neutrophil’s inflammatory response and are involved in the chemotaxis and degranulation(Futosi and Mócsai 2016). fMLP’s anti-tumor activities include macrophage activation and the induction of lysozyme release and pro-inflammatory factors (Liao et al. 2022), aiding in tumor cell demise. Moreover, it influences white blood cells and macrophages, impacting tumor growth, spread, and mobility (Dahlgren et al. 2016; Netea-Maier et al. 2018). However, there is no evidence to suggest that fMLP does help radiation-induced mutant cells to survive. Beyond its role in immunity, fMLP has demonstrated potential in combating HIV-1 infection by suppressing the expression and activity of CCR5 (Karakaya et al. 2021), a vital co-receptor for HIV-1 entry into CD4 + T cells (Rossi et al. 2011). The susceptibility of organisms to physical or chemical alterations induced by irradiation varies widely, a concept known as radiation sensitivity (McKenna and Muschel 2003; Seibold et al. 2020). This study uncovered the intriguing radioprotective properties of fMLP, an agent that mitigates radiation damage.

In this work, we focused on the effect and mechanism of fMLP administration on irradiation-induced hematopoietic and intestinal systems injury. The CCK-8 assay affirmed fMLP’s minimal toxicity and its notable ability to reverse radiation-induced apoptosis. The impact of irradiation on organs differs due to the diverse radiation sensitivity of cells within tissues. The bone marrow hematopoietic system, spleen, and intestine are particularly susceptible, yet fMLP effectively protects peripheral white blood cells and maintains spleen integrity. Furthermore, examination of intestinal tissues revealed that fMLP encourages cellular regeneration and minimizes apoptosis, thereby reversing intestinal damage. There were no obvious indications on radiation -induced mutations that may arise or give rise to tumorigenesis. We are looking forward to focusing on this topic and revealing its role in possible mutagenesis and tumor development.

The efficacy of fMLP in enhancing the survival rate of irradiated mice was noted to surpass that of WR-2721, although it paled in comparison to LPS treatment. This outcome seems paradoxical, given the generally held understanding of LPS as a more toxic agent than fMLP. The discrepancy may be attributed to the superior solubility of LPS within the biological setting. The degree of protection afforded by fMLP against cellular apoptosis was dose-dependent. At an irradiation dose of 6 Gy, the apoptosis rate of BMCs treated with fMLP showed no significant difference from the control group. However, a distinct reduction in apoptosis was observed at 12 Gy, suggesting a dose-response relationship. This trend was consistent in both BMCs and HIECs, which implied the radioprotective capabilities of fMLP were more robust against high radiation doses. Accumulated data indicate that fMLP can play an important role in apoptotic homeostasis through physiologically relevant nitric oxide (NO) levels (Porro et al. 2019). Mitochondrial injury leads to the activation of mitochondrial apoptosis pathway with the involvement of the Bcl-2 family members (Wang 2014). The release of NO from fMLP treated neurons induces apoptosis through the mitochondrial pathway and modulation of the Bcl-2 family proteins (Manucha 2017). It may be of interest to explore the effects of fMLP on mitochondria after irradiation. In this study, we paid more attention on acute toxicity of fMLP in vivo and in vitro, because high-dose irradiation always mediates acute injury. However, the long-term survival of animals after administration of fMLP should be considered in future studies.

To unravel the underlying radioprotective mechanism of fMLP, RNA sequencing was employed to analyze gene expression patterns in spleen cells. The results revealed 593 differentially expressed genes, comprising 343 upregulated and 250 downregulated genes. Upregulated genes were significantly enriched in functions related to the cell cycle, cell adhesion, and immune response pathways. Furthermore, GO and KEGG analyses pointed towards innate immunity and cell cycle processes as the most affected biological systems. This suggested that fMLP might exert its radioprotective effect by modulating the cell cycle and enhancing the body’s innate immune response. This hypothesis was supported by literature reports associating innate immunity with radiation protection and observing IR-induced alterations in the cell cycle, particularly during DNA synthesis and the G2 phase. Experimental assays conducted on HIEC cells confirmed that fMLP increased the population of cells in the G0/G1 phase, indicating a regulatory role in cell cycle progression. The protein expression analysis revealed that fMLP mitigated IR-induced increases in P21 and P27 levels while enhancing Cdk2 protein levels. The literature consistently highlights the crucial role of P21 as an intermediary in arresting the cell cycle in response to DNA damage. Specifically, P21 is a key regulator of cell cycle progression, primarily functioning as a cyclin-dependent kinase inhibitor (CDKI) during the G1 phase. Through its binding to CDK2, P21 represses cell cycle progression by blocking the transition of proliferating cells from G1 to S phase, thereby preventing further DNA replication and allowing for DNA repair mechanisms to take place (Amani et al. 2021). Therefore, fMLP’s protective mechanism involves mitigating cell cycle arrest, thereby minimizing radiation-induced cellular damage. Moreover, fMLP induced intracellular reactive oxygen species (ROS) production, caspase activation as well as DNA fragmentation in human leukocytes (Espino et al. 2011). The induction of radiation-induced DNA damage in the presence of fMLP will be considered in future studies.

## Conclusion

Our research delved into the protective effects of fMLP against IR-induced damage in the hematopoietic and intestinal systems and elucidated the underlying mechanisms. Results from in vivo experiments revealed that fMLP increased the survival rate of mice exposed to total body irradiation. Notably, it offered significant protection against radiation damage in the intestinal system. At the cellular level, fMLP mitigated IR-induced cell cycle arrest, as evidenced by an increase in the number of cells progressing into the G0/G1 phase. This effect was further confirmed through protein expression analysis, showing altered levels of P21, P27, and Cdk2. Collectively, fMLP provides radioprotection to both the hematopoietic and intestinal systems by mitigating cell cycle disruptions caused by IR exposure.

## Data Availability

No datasets were generated or analysed during the current study.

## References

[CR1] Amani J, Gorjizadeh N, Younesi S, et al. Cyclin-dependent kinase inhibitors (CDKIs) and the DNA damage response: the link between signaling pathways and cancer. DNA Repair (Amst). 2021;102:103103. 10.1016/j.dnarep.2021.103103 33812232 10.1016/j.dnarep.2021.103103

[CR2] Bedouhène S, Dang PM, Hurtado-Nedelec M, El-Benna J. Neutrophil degranulation of azurophil and specific granules. Methods Mol Biol. 2020;2087:215–22. 10.1007/978-1-0716-0154-9_16 31728994 10.1007/978-1-0716-0154-9_16

[CR3] Cavaillon JM. Exotoxins and endotoxins: inducers of inflammatory cytokines. Toxicon. 2018;149:45–53. 10.1016/j.toxicon.2017.10.016 29056305 10.1016/j.toxicon.2017.10.016

[CR4] Dahlgren C, Gabl M, Holdfeldt A, Winther M, Forsman H. Basic characteristics of the neutrophil receptors that recognize formylated peptides, a danger-associated molecular pattern generated by bacteria and mitochondria. Biochem Pharmacol. 2016;114:22–39. 10.1016/j.bcp.2016.04.014 27131862 10.1016/j.bcp.2016.04.014

[CR5] Espino J, Bejarano I, Paredes SD, Barriga C, Rodríguez AB, Pariente JA. Protective effect of melatonin against human leukocyte apoptosis induced by intracellular calcium overload: relation with its antioxidant actions. J Pineal Res. 2011;51(2):195–206. 10.1111/j.1600-079X.2011.00876.x 21470303 10.1111/j.1600-079X.2011.00876.x

[CR6] Futosi K, Mócsai A. Tyrosine kinase signaling pathways in neutrophils. Immunol Rev. 2016;273(1):121–39. 10.1111/imr.12455 27558332 10.1111/imr.12455

[CR7] Ghita M, Dunne V, Hanna GG, Prise KM, Williams JP, Butterworth KT. Preclinical models of radiation-induced lung damage: challenges and opportunities for small animal radiotherapy. Br J Radiol. 2019;92(1095):20180473. 10.1259/bjr.20180473 30653332 10.1259/bjr.20180473 PMC6541188

[CR8] Hegyesi H, Sándor N, Sáfrány G, Lovas V, Kovács Á, Takács A, Kőhidai L, Turiák L, Kittel Á, Pálóczi K, et al. Radio-detoxified LPS alters bone marrow-derived extracellular vesicles and endothelial progenitor cells. Stem Cell Res Ther. 2019;10(1):313. 10.1186/s13287-019-1417-4 31665090 10.1186/s13287-019-1417-4 PMC6819448

[CR9] Karakaya B, van Moorsel CHM, Veltkamp M, Roodenburg-Benschop C, Kazemier KM, van der Helm AHM, et al. A polymorphism in C-C chemokine receptor 5 (CCR5) associates with Löfgren’s syndrome and alters receptor expression as well as functional response. Cells. 2021;10(8):1967. 10.3390/cells10081967 34440736 10.3390/cells10081967 PMC8394428

[CR10] Kilic AK, Esendagli G, Sayat G, Talim B, Karabudak R, Kurne AT. Promotion of experimental autoimmune encephalomyelitis upon neutrophil granulocytes’ stimulation with formyl-methionyl-leucyl-phenylalanine (fMLP) peptide. Autoimmunity. 2015;48(6):423–8. 10.3109/08916934.2015.1030615 25826286 10.3109/08916934.2015.1030615

[CR11] Lai L, Shin GY, Qiu H. The role of cell cycle regulators in cell survival-dual functions of cyclin-dependent kinase 20 and p21Cip1/Waf1. Int J Mol Sci. 2020;21(22):8504. 10.3390/ijms21228504 33198081 10.3390/ijms21228504 PMC7698114

[CR12] Liao HR, Kao YY, Leu YL, Liu FC, Tseng CP. Larixol inhibits fMLP-induced superoxide anion production and chemotaxis by targeting the βγ subunit of Gi-protein of fMLP receptor in human neutrophils. Biochem Pharmacol. 2022;201:115091. 10.1016/j.bcp.2022.115091 35569521 10.1016/j.bcp.2022.115091

[CR13] Liu Z, Lei X, Li X, Cai JM, Gao F, Yang YY. Toll-like receptors and radiation protection. Eur Rev Med Pharmacol Sci. 2018;22(1):31–9. 10.26355/eurrev_201801_14097 29364499 10.26355/eurrev_201801_14097

[CR14] Maddika S, Ande SR, Panigrahi S, Paranjothy T, Weglarczyk K, Zuse A, et al. Cell survival, cell death and cell cycle pathways are interconnected: implications for cancer therapy. Drug Resist Updat. 2007;10(1–2):13–29. 10.1016/j.drup.2007.01.003 10.1016/j.drup.2007.01.003 17303468

[CR15] Manucha W. Mitochondrial dysfunction associated with nitric oxide pathways in glutamate neurotoxicity. Clin Investig Arterioscler. 2017;29(2):92–7. 10.1016/j.arteri.2016.04.002 10.1016/j.arteri.2016.04.002 27240721

[CR16] McKenna WG, Muschel RJ. Targeting tumor cells by enhancing radiation sensitivity. Genes Chromosomes Cancer. 2003;38(4):330–8. 10.1002/gcc.10296 14566853 10.1002/gcc.10296

[CR17] Netea-Maier RT, Smit JWA, Netea MG. Metabolic changes in tumor cells and tumor-associated macrophages: a mutual relationship. Cancer Lett. 2018;413:102–9. 10.1016/j.canlet.2017.10.037 29111350 10.1016/j.canlet.2017.10.037

[CR18] Polesskaya O, Wong C, Lebron L, Chamberlain JM, Gelbard HA, Goodfellow V, et al. MLK3 regulates fMLP-stimulated neutrophil motility. Mol Immunol. 2014;58(2):214–22. 10.1016/j.molimm.2013.11.016 24389043 10.1016/j.molimm.2013.11.016 PMC3946811

[CR19] Porro C, Cianciulli A, Trotta T, Lofrumento DD, Calvello R, Panaro MA. Formyl-methionyl-leucyl-phenylalanine induces apoptosis in murine neurons: evidence for NO-dependent caspase-9 activation. Biology (Basel). 2019;8(1):4. 10.3390/biology8010004 30621183 10.3390/biology8010004 PMC6466069

[CR20] Prăvălie R, Bandoc G. Nuclear energy: between global electricity demand, worldwide decarbonisation imperativeness, and planetary environmental implications. J Environ Manage. 2018;209:81–92. 10.1016/j.jenvman.2017.12.043 29287177 10.1016/j.jenvman.2017.12.043

[CR21] Rossi R, Lichtner M, De Rosa A, Sauzullo I, Mengoni F, Massetti AP, et al. In vitro effect of anti-human immunodeficiency virus CCR5 antagonist maraviroc on chemotactic activity of monocytes, macrophages and dendritic cells. Clin Exp Immunol. 2011;166(2):184–90. 10.1111/j.1365-2249.2011.04409.x 21985364 10.1111/j.1365-2249.2011.04409.x PMC3219893

[CR22] Roy A, Banerjee S. p27 and leukemia: cell cycle and beyond. J Cell Physiol. 2015;230(3):504–9. 10.1002/jcp.24819 25205053 10.1002/jcp.24819

[CR23] Seibold P, Auvinen A, Averbeck D, Bourguignon M, Hartikainen JM, Hoeschen C, et al. Clinical and epidemiological observations on individual radiation sensitivity and susceptibility. Int J Radiat Biol. 2020;96(3):324–39. 10.1080/09553002.2019.1665209 31539290 10.1080/09553002.2019.1665209

[CR24] Simpson BW, Trent MS. Pushing the envelope: LPS modifications and their consequences. Nat Rev Microbiol. 2019;17(7):403–16. 10.1038/s41579-019-0201-x 31142822 10.1038/s41579-019-0201-x PMC6913091

[CR25] Wang K. Molecular mechanisms of hepatic apoptosis. Cell Death Dis. 2014;5(1):e996. 10.1038/cddis.2013.499 24434519 10.1038/cddis.2013.499 PMC4040708

[CR26] Wei J, Wang B, Wang H, Meng L, Zhao Q, Li X, et al. Radiation-induced normal tissue damage: oxidative stress and epigenetic mechanisms. Oxid Med Cell Longev. 2019;2019:3010342. 10.1155/2019/3010342 31781332 10.1155/2019/3010342 PMC6875293

[CR27] Wille C, Eiseler T, Langenberger ST, Richter J, Mizuno K, Radermacher P, et al. PKD regulates actin polymerization, neutrophil deformability, and transendothelial migration in response to fMLP and trauma. J Leukoc Biol. 2018;104(3):615–30. 10.1002/jlb.4a0617-251rr 29656400 10.1002/jlb.4a0617-251rr

[CR28] Xie LW, Cai S, Zhao TS, Li M, Tian Y. Green tea derivative (-)-epigallocatechin-3-gallate (EGCG) confers protection against ionizing radiation-induced intestinal epithelial cell death both in vitro and in vivo. Free Radic Biol Med. 2020;161:175–86. 10.1016/j.freeradbiomed.2020.10.012 33069855 10.1016/j.freeradbiomed.2020.10.012

[CR30] Yang KH, Fang H, Ye JS, Gong JZ, Wang JT, Xu WF. The main functions and structural modifications of tripeptide N-formyl-methionyl-leucyl-phenylalanine (fMLP) as a chemotactic factor. Pharmazie. 2008;63(11):779–83. 19069235

[CR29] Yang H, Clarke JL, Thompson JR. Nuclear energy: improve collaboration. Science. 2016;353(6304):1107. 10.1126/science.aai8681 27609880 10.1126/science.aai8681

